# Remodeling of Hyperpolarization-Activated Current, I_h_, in Ah-Type Visceral Ganglion Neurons Following Ovariectomy in Adult Rats

**DOI:** 10.1371/journal.pone.0071184

**Published:** 2013-08-12

**Authors:** Guo-Fen Qiao, Zhao Qian, Hong-Li Sun, Wen-Xiao Xu, Zhen-Yu Yan, Yang Liu, Jia-Ying Zhou, Hao-Cheng Zhang, Li-Juan Wang, Xiao-Dong Pan, Yili Fu

**Affiliations:** 1 State Key Laboratory of Robotics and System, Harbin Institute of Technology, Harbin, Heilongjiang, China; 2 Department of Pharmacology, Harbin Medical University, Harbin, Heilongjiang, China; 3 Department of Pharmacology, Da-Qing Campus of Harbin Medical University, Da-Qing, Heilongjiang, China; 4 Department of Orthopedics, the First Affiliated Hospital, Harbin Medical University, Harbin, Heilongjiang, China; Dalhousie University, Canada

## Abstract

Hyperpolarization-activated currents (I_h_) mediated by hyperpolarization-activated cyclic nucleotide-gated (HCN) channels modulate excitability of myelinated A− and Ah-type visceral ganglion neurons (VGN). Whether alterations in I_h_ underlie the previously reported reduction of excitability of myelinated Ah-type VGNs following ovariectomy (OVX) has remained unclear. Here we used the intact nodose ganglion preparation in conjunction with electrophysiological approaches to examine the role of I_h_ remodeling in altering Ah-type neuron excitability following ovariectomy in adult rats. Ah-type neurons were identified based on their afferent conduction velocity. Ah-type neurons in nodose ganglia from non-OVX rats exhibited a voltage ‘sag’ as well as ‘rebound’ action potentials immediately following hyperpolarizing current injections, which both were suppressed by the I_h_ blocker ZD7288. Repetitive spike activity induced afterhyperpolarizations lasting several hundreds of milliseconds (termed post-excitatory membrane hyperpolarizations, PEMHs), which were significantly reduced by ZD7288, suggesting that they resulted from transient deactivation of I_h_ during the preceding spike trains. Ovariectomy reduced whole-cell I_h_ density, caused a hyperpolarizing shift of the voltage-dependence of I_h_ activation, and slowed I_h_ activation. OVX-induced I_h_ remodeling was accompanied by a flattening of the stimulus frequency/response curve and loss of PEMHs. Also, HCN1 mRNA levels were reduced by ∼30% in nodose ganglia from OVX rats compared with their non-OVX counterparts. Acute exposure of nodose ganglia to 17beta-estradiol partly restored I_h_ density and accelerated I_h_ activation in Ah-type cells. In conclusion, I_h_ plays a significant role in modulating the excitability of myelinated Ah-type VGNs in adult female rats.

## Introduction

Gender differences in blood pressure have been known for decades [1]–[4]. Blood pressure in premenopausal women is significantly lower compared with men of the same age [5], but it increases to male levels in postmenopausal women [6]. Studies in rats have demonstrated a significant increase in blood pressure following surgical ovariectomy, suggesting a role of female hormones in mediating the hypotensive effect. [7]. However, the exact mechanisms underlying this change in blood pressure are still not fully understood. Recently, our combined electrophysiological/neuroanatomical studies [8], [9] identified a gender-specific population of myelinated Ah-type visceral ganglion neurons (VGN) in adult female rats, including baroreceptor neurons in nodose ganglia. This unique population of Ah-type VGN displayed increased excitability, suggesting the possibility that they are part of a feed-back regulatory mechanism responsible for maintaining low blood pressure. Although the density of this unique Ah-type population did not change following bilateral ovariectomy (OVX), their excitability was significantly reduced. Acute exposure to physiological concentrations of 17beta-estradiol (17beta-E_2_) restored excitability via activation of the GPR30 receptor pathway [10]. Intriguingly, similar electrical remodeling was not observed in myelinated A− or unmyelinated C-type VGNs in adult female ovariectomized rats in the same study.

The neuronal excitability of sensory ganglion neurons (VGN) is majorly determined by the activity of voltage-gated Na^+^ channels [11], large- and small-conductance Ca^2+^-activated K^+^ channels (BK, SK) [12], and hyperpolarization-activated cyclic nucleotide-gated (HCN) channels [13]. Here, we focus on the role of HCN-mediated hyperpolarization-activated inward current, I_h_, because 1) I_h_ is a key regulator of neuronal firing properties. 2) All subtypes of HCN channels (HCN1 to HCN4) are functionally expressed in nodose neurons [14], but the HCN1 subtype is only observed in myelinated afferents [15]. 3) I_h_ density is 5–20-times larger in myelinated afferents compared with unmyelinated C-type VGNs [15]. I_h_ also exhibits faster activation in A− and Ah-type cells compared with C-type cells. 4) Excitability of sensory neurons is reduced by the I_h_ blocker ZD7288 [13], [16]–[18]. 5) I_h_ undergoes remodeling under certain physiological [19], [20] and pathophysiological conditions, such as pain [17], [18], diabetes [14], mental disorder such as impaired motor learning and memory [21] and anxiolytic- and antidepressant-like behaviors by knockdown HCN1 [22], and baroreceptor dysfunction [23]. 6) Ah-types VGNs have wider discharge capability and lower firing thresholds [8], [9]. In addition, Ah-types, but not A− or C-type neurons, were shown to exhibit reduced excitability following OVX [10]. Taken all data together, we examined the possibility that ovariectomy alters the density and/or biophysical properties of I_h_ in Ah-type VGNs, and that these changes are associated with altered excitability.

The present data demonstrate that I_h_ was expressed in all three VGN subtypes identified via their afferent conduction velocity (CV) using the intact nodose slice preparation of adult female rats. Following OVX, the excitability of Ah-type neurons was reduced, concomitant with a down-regulation of I_h_ density and a shift of the I_h_ activation curve to more negative potentials. The level of HCN1 mRNA was significantly reduced in nodose ganglia from OVX animals. I_h_ density was partially restored in Ah-type neurons when acutely exposed to 17beta-E_2_.

## Materials and Methods

Standard electrophysiological whole-cell patch recordings were conducted under the current- or voltage-clamp configuration to investigate the effect of ovariectomy on HCN channel-mediated currents (I_h_) in vagal nodose ganglion neurons from adult rats. Specifically, the nodose ganglion slice preparation was utilized to examine the role of ovariectomy-induced I_h_ remodeling in altering the electrical properties of nodose ganglion neurons, including CV, excitability and discharge properties [24], [25].

### Experimental Animals

Adult female *Sprague-Dawley* (SD) rats (220–250 g, at least 14 weeks of age) were directly purchased from Wei Tong Li Hua Experimental Animal Technology Co, Ltd, Beijing, China, with SPF grade and licensed under SCXK (Beijing) 2012–0001. All rats were maintained at the animal facility of the Second Affiliated Hospital of Harbin Medical University with a 12/12 hour light cycle for 3 days before they were used for experiments. All animal use protocols were pre-approved by the Institutional Animal Care and Use Committees of the School of Medical Science, Harbin Medical University, China.

### Vagal Slice Preparation

Adult female Sprague-Dawley rats (*n* = 28, 220–250 g, at least 14 weeks of age) were used for the preparation of nodose ganglion slices to study myelinated A− and Ah− VGN as well as unmyelinated C-type VGN [24], [25]. The bilateral dissection of the vagal ganglia each with ∼2.0 cm of their vagus nerve attached, ganglia slicing, and enzymatic digestion have been described elsewhere [25]. Briefly, the rats were placed in an airtight induction chamber for inhalation of the anesthetic Metofane (Methoxyflurane, Schering-Plough Animal Health Corp., NJ, USA). Upon lack of reflex response to tail pinch the animals were immediately sectioned at the mid-auxiliary region, preserving at least 2 cm of the vagus nerve. Surgical dissection of the vagal ganglia was performed under a stereomicroscope (×40). The vagal ganglia were placed immediately in chilled recording solution (composition see below) and sliced following removal of all connective tissue surrounding the ganglia and nerves. We used either a vibrating blade microtome (Leica, VT1000s) to section the agarose-embedded nodose ganglion or trimmed the un-embedded ganglion manually using a scalpel. The slices were then transferred to a Petri dish filled with 5.0 ml of Earls Balanced Salt Solution (Sigma) containing 1.0 mg/ml of type-II collagenase (Worthington Biochemical, Lakewood, NJ, USA) and incubated at 37°C for 45 min followed by fresh support medium with 5.0 mg/ml trypsin (Worthington Biochemical, Lakewood, NJ, USA) at 37°C for an additional 25 min. The enzyme solution was then repeatedly replaced with chilled (∼4°C) recording solution prior to transferring to the recording chamber. The preparation was positioned with the enzyme-exposed neurons facing up and gently held in place with a tissue anchor.

All electrophysiological measurements were performed utilizing the ruptured patch technique (see below). Neurons were visualized using an upright microscope (Axioskop, Zeiss), and 100 W Halogen light source with an infrared bandpass filter placed in the light path. Using an infrared-sensitive CCD camera (Hamamatsu) under the control of a video image processor (Hamamatsu) it was possible to clearly visualize neurons at depths approaching 150 microm. A positive pressure was applied to the pipette interior while approaching target cell and released upon cell contact. A Giga-ohm seal was formed by applying a negative pressure to the pipette interior.

### Surgical Ovariectomy

Bilateral ovariectomy was performed from a dorso-lateral approach [26]. Rats were anesthetized with a combination of xylazine (10 mg/kg) and ketamine (75 mg/kg) given intraperitoneally. Anesthetized animals were placed in a lateral position and both flanks were shaved (anatomic landmark of the ovaries: caudal end of the rib cage). The shaved area was cleaned using chlorhexidine scrub and disinfected with 70% ethanol and povidone-iodine (7.5%). A 2.0-cm incision was made on the left lateral side along a line spanning from the 2^nd^ to the 5^th^ lumbar vertebra, using a scalpel blade. The left ovary and associated fat were located and externalized by gentle retraction. After removal of the ovary, the peritoneal cavity, muscle layers, and skin were closed successively with 3-0 absorbable sutures. The same procedure was repeated for removal of the right ovary. After recovering from anesthesia, the animals were monitored for at least 30 min to ensure that there was no bleeding from the surgery, and then were returned to the animal facility. Animals in the sham group were subjected to the same anesthesia and surgery protocol, but did not undergo ovariectomy. Electrophysiological measurements in Ah-type neurons in nodose ganglia slices isolated from control, ovariectomized, and sham operated animals revealed no significant differences, whereas some electrophysiological parameters in the OVX group significantly differed from their respective values in both the control and sham group (see Table S1). These results support the notion that electrophysiological alterations seen in the OVX group result from excision of the ovaries rather than from the surgical procedure *per se.* All experiments were therefore performed on preparations isolated from control and OVX rats. Four weeks after ovariectomy, the animals were sacrificed for experimental use.

### Vagal Stimulation

As described previously [24], [25], nodose ganglion slices with their vagus nerves attached were transferred to the recording chamber. A tissue harp (Warner Instruments, CT, and USA) was used to hold the preparation in a stable position during continuous superfusion. A bipolar stimulation electrode was placed at the amputated end of the vagus nerve at a distance ≥15 mm from the intraganglionic recording site, enabling sufficient separation of the stimulus and response signal for accurate CV measurements under our experimental conditions (∼22°C). Short duration (≤200 micros) monophasic constant-current pulses were applied for vagal stimulation. To examine the frequency response of the preparation, a series of 1-s episodes of repetitive electrical stimuli at frequencies ranging from 1 to 100 Hz was applied to each of the three fiber types (Master-8, the 8-channel programmable Pulse Generator, Jerusalem, Israel) and the electrical responses were recorded in their respective soma.

### Electrophysiological Solutions

For action potential (AP) recordings, the composition of the intracellular solution was (in mmol/L): NaCl 10; KCl 50; K_2_SO_4_ 50; MgCl_2_ 5; HEPES 10, pH adjusted to 7.25 using 1 N KOH. Immediately prior to filling the patch pipettes, 2.0 mmol/L Mg-ATP and 2.0 mmol/L Na-GTP (both Sigma) were added to the pipette solution, along with 4.0 mmol/L BAPTA-Na and 0.25 mmol/L CaCl_2_ for a final buffered [Ca^2+^] of 100 nmol/L. The composition of the extracellular recording solution was (in mmol/L): NaCl 137; KCl 5.4; MgCl_2_ 1.0; CaCl_2_ 2.0; glucose 10; HEPES 10, pH adjusted to 7.30–7.35 using 1.0 N NaOH. The osmolarity of the extracellular and intracellular solutions was adjusted to 310–315 and 290–295 mmol/kg, respectively, using D-manitol (Sigma).

### Drugs and Chemicals

A 1.0-mM stock solution of the HCN channel antagonist ZD7288 (Tocris, Ellisville, MO, USA) was prepared in extracellular recording solution and kept at 4°C until use. The ZD7288 stock solution was further diluted 100- to 1,000-fold immediately before use. 17beta-estradiol (1.0 microM) stock solution (Sigma, St Luis, MO, USA) was prepared freshly and stored at -20°C protected from light. The preparation was continuously superfused (1 ml/min) with bath solution containing either ZD7288 or 17beta-estradiol for at least 10 min prior to data collection to ensure steady-state effects of these agents.

### Electrophysiological Techniques

Whole-cell patch recordings were performed using the Axopatch 700B amplifier (Axon Instruments, Union City, CA, USA). Borosilicate glass pipettes (Sutter Instruments, Novato, CA, USA) were pulled and polished down to 1.5–2.4 Mega-ohm, as measured in AP recording solution (see below). Following the alpha-ion of a Giga-ohm seal, the pipette capacitance was compensated. The total cell capacitance (60–90 pF) and electrode access resistance (3–5 Mega-ohm) were also compensated generally to within 20–40%. All patch experiments were conducted at room temperatures (22–23°C). Data traces were low pass filtered to 10 kHz and digitized at 50 kHz using pCLAMP 10.2 (Axon Instruments, Union City, CA, USA) and Digidata 1440A (Molecular Devices, Sunnyvale, CA, USA) operating on a PC platform.

For current-clamp experiments, five distinct experimental protocols were performed. *First*, to classify the type of neuron being examined, vagal electrical stimulation-evoked action potentials (AP) were recorded and conduction velocity (CV) was determined; s*econd*, to measure the frequency response of the preparation, successive 1-s episodes of repetitive electrical stimulation at increasing rates (range: 1 to 100 s^-1^) were applied to the vagus nerve and the electrical responses in the soma of each of the three neuronal subtypes were recorded; *third*, once the neuronal subtype was identified based on CV properties, the membrane potential of the impaled cell was hyperpolarized using current magnitudes ranging from −80 to −120 pA for 400 ms, to quantify the magnitude and kinetics of the hyperpolarization-induced depolarizing voltage sag (DVS) as well as to examine the occurrence of rebound action potentials upon cessation of the hyperpolarizing current; *fourth*, a single somatic AP was elicited by applying a brief (≤2.0 ms) suprathreshold depolarizing current pulse through the patch electrode, to measure upstroke velocity (dV/dt) and action potential duration (APD); *fifth*, depolarizing 1-s current pulses (50–300 pA) were applied through the patch electrode from a holding potential of −60 mV, to determine neuronal excitability. This adjustment from the cell’s intrinsic resting membrane potential was necessary to account for intercellular differences in resting potential which in turn would give rise to differences in the availability of a number of membrane conductance and confounding comparisons between cells. For voltage-clamp experiments, a two-step voltage protocol composed of an initial 1-s hyperpolarizing step from −40 mV to −130 mV (in 5-mV steps) followed by a 600-ms step to −80 mV was applied at 1-s intervals. Holding potential was −40 mV.

### Quantitative Real-time PCR

RNA was extracted from rat nodose ganglia using TRIZOL reagent. A 0.5-microgram RNA aliquot was transcribed to its cDNA using random primers and the High Capacity cDNA Reverse Transcription Kit (AB Applied Biosystems, USA). To detect the level of HCN1 mRNA, the generated cDNA was used in quantitative real-time PCR, using specific primers for the chromosomal genes HCN1. The mRNA levels were quantified by SYBR Green incorporation on ABI 7500 fast Real Time PCR system (Applied Biosystems, USA). GAPDH was used as an internal control. The Real-time PCR primer sequences for HCN1 were forward: 5′- ACATGCTGTGCATTGGTTATGGCG-3′and reverse: 5′- GACAAACATGGCATAGCAGGTGGC-3′.The sequences of GAPDH primers were forward primer: 5′-TCTACATGTTCCAGTATGACTC-3′and reverse: 5′-ACTCCACGACATACTCAGCACC-3'. And the ^ΔΔ^Ct method provides the relative gene expression levels by averaging cycle threshold (Ct) values from triplicate RT-PCR reaction for the target and housekeeping genes.

### Data Analysis

Pooled statistics were calculated using Excel (Microsoft, Bellevue, WA, USA) or SPSS 13.0 software with measures expressed as the mean ±1 SD. Comparisons across population samples were performed using either Student’s *t*-test or ANOVA followed by a post hoc *t*-test where appropriate. Data populations exhibiting an overlap of 5% or less were considered to be significantly different.

## Results

### Frequency- and Hyperpolarization-dependent Post-excitatory Membrane Hyperpolarization in A-type Visceral Ganglion Neurons from Adult, Non-ovariectomized Rats

**Figure 1:**
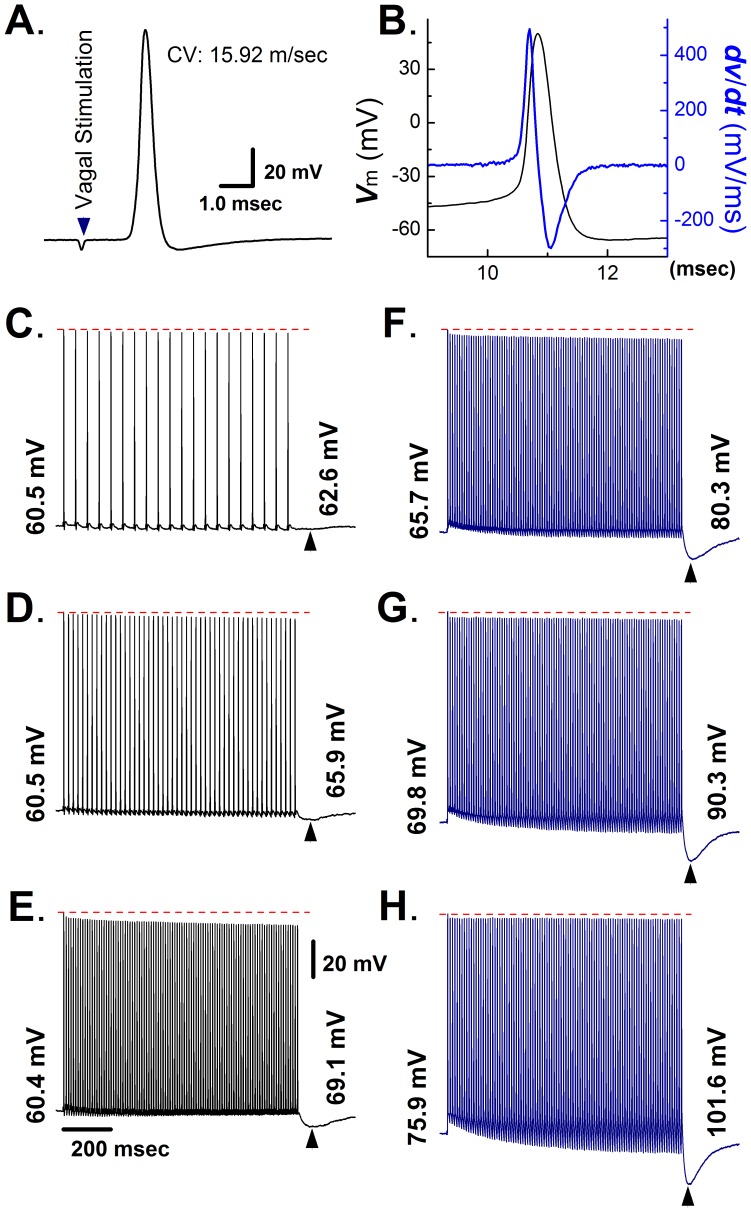
Electrical properties of myelinated A-type vagal ganglion neurons in the nodose ganglion preparation of adult non-ovariectomized rats. **A**) Vagal stimulation-evoked transmembrane action potential in a myelinated A-type vagal ganglion neuron (VGN). The value for the conduction velocity (CV) measured between the stimulation and recording site was indicative of an A-type cell. **B**) Transmembrane action potential recorded from the same neuron as in (**A**) and its first derivative over time (blue trace). **C**) – **E**) Post-excitatory membrane hyperpolarization (PEMH) following 1-s trains of vagal stimulation at 20 (**C**), 50 (**D**), and 100 Hz (**E**). Numbers at the end of each trace denote most negative value of the membrane potential during each PEMH. **F**) – **H**) PEMHs induced by 1-s trains of vagal stimulation at 100 Hz superimposed on different degrees of RMP hyperpolarization. Hyperpolarizing currents were applied throughout the recordings, including the post-tetanic period. Numbers at the beginning and end of each trace denote RMP and most negative membrane potential value during PEMH, respectively. Scale bars in (**E**) also apply to (**C**) – (**H**).

In this study, myelinated A-type VGNs from intact nodose ganglion slices were identified based on their CV and action potential properties. Representative examples of A-type transmembrane action potentials evoked by vagal nerve stimulation and direct current injection into the soma are shown in Figure 1A and B, respectively. Values for CV (15.9 m/s), AP firing threshold (−50.2 mV), maximal upstroke velocity (436 V/s), and AP duration at 50% repolarization (0.68 ms) as well as the absence of a repolarization ‘hump’ are all indicative of an A-type VGN [9]. Further, A-type neurons were found to exhibit short afterhyperpolarizations and a stable 1∶1 stimulus/response pattern during 1-s electrical stimulation of the vagus nerve at frequencies ranging from 1 to 100 Hz. The peak amplitude of evoked action potentials progressively decreased during electrical stimulation, with the effect being more pronounced at higher stimulation rates (Figure 1C–E). Notably, the termination of each stimulus train was followed by a prolonged afterhyperpolarization (designated hereafter as post-excitatory membrane hyperpolarization, PEMH), which typically peaked within <100 ms upon termination of vagal stimulation and resolved within ∼ 200 msec. The peak amplitude of the PEMH increased with the number of action potentials used to provoke it (Figure 1C–E). Sustained hyperpolarizations of the RMP via constant current injection during rapid vagal stimulation exerted three distinct effects on the electrical behavior of A-type neurons, as shown in Figure 1 F–H. First, it attenuated the progressive decrease in spike amplitude, possibly owing to removal of slow inactivation of fast, voltage-gated sodium channels [27], [28]. Second, it caused the afterhyperpolarization following each action potential during the train to become progressively more negative (see Figure S1 for an expanded time scale of the initial portion of the recording shown in Figure 1H). Third, the peak PEMH amplitude gradually increased with the degree of RMP hyperpolarization. Importantly, the action potential remained largely unchanged during repetitive vagal stimulation.

**Figure 2:**
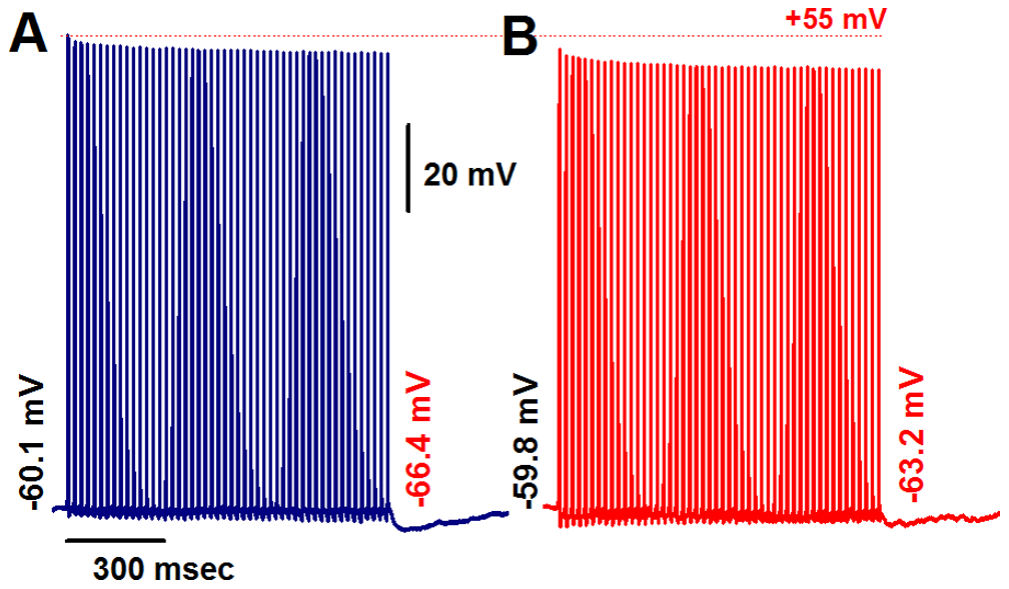
The I_h_ blocker ZD7288 reduces peak PEMH amplitude in A-type VGNs. In the absence of ZD7288, a 1-s train of vagal stimulation at 50 Hz causes a PEMH. (**A**). Exposure to 1 microM ZD7288 causes no change in the stimulus/response pattern but markedly reduces the peak PEMH amplitude. Numbers at the beginning and end denote RMP and most negative membrane potential during PEMH, respectively. (**B**). Scale bars in (**A**) also apply to (**B**).

We next examined if the PEMH evoked by repetitive stimulation was mediated by the transient deactivation of hyperpolarization and cyclic nucleotide-activated (HCN) channels that underlie I_h_. Application of the I_h_ channel blocker ZD7288 (1 microM) did not significantly alter RMP or the stimulus-response pattern in an A-type neuron, but eliminated the progressive hyperpolarization during repetitive stimulation and markedly reduced peak PEMH amplitude (Figure 2). On average, ZD7288 application did not significantly alter RMP (control: −59.8±4.6 mV, ZD7288: −60.4±4.8 mV; *n* = 8; *P*>0.05 by paired *t*-test) but significantly reduced peak PEMH amplitude (control: −6.26±1.24 mV, ZD7288: −2.89±0.74 mV; *P*<0.01 by paired *t*-test).

### C-type Visceral Ganglion Neurons from Adult, Non-ovariectomized Rats Lack PEMHs

We also examined the electrical properties of unmyelinated C-type VGNs. A representative example is shown in Figure S2. C-type neurons exhibited lower CVs (Figure S1A), less negative threshold potentials, slower AP upstroke velocities, and longer APD (Figure S2B) with a significant repolarization ‘hump’ compared with A-type neurons under identical experimental conditions. Also, a stable 1∶1 stimulus/response ratio during vagal stimulation could only be maintained up to a stimulation frequency of 20 Hz, without inducing PEMHs (Figure S2C and D).

### I_h_ in Myelinated Ah-type Visceral Ganglion Neurons from Adult, Non-ovariectomized Rats

**Figure 3:**
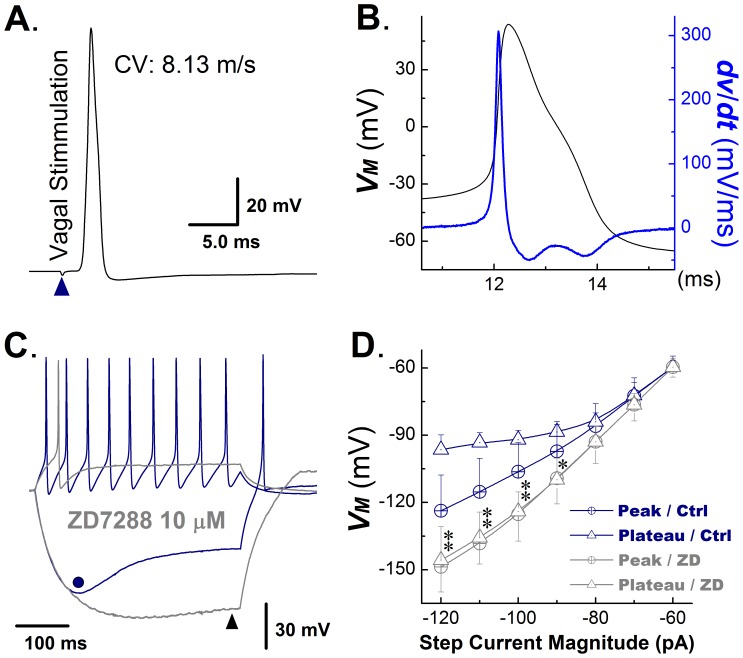
I_h_ in myelinated Ah-type VGNs from adult, non-ovariectomized rats. **A**) Vagal stimulation-evoked transmembrane action potential in a myelinated Ah-type vagal ganglion neuron (VGN). The value for the conduction velocity (CV) measured between the stimulation and recording site was indicative of an Ah-type cell. **B**) Transmembrane action potential recorded from the same neuron as in (**A**) and its first derivative over time (blue trace). Note the presence of a repolarization ‘hump’. **C**) Examples of membrane potential responses of an Ah-type VGN to a depolarizing and a hyperpolarizing current injection step. The cell was first injected with 150 pA current step, giving rise to repetitive action potential firing, which ceased upon termination of the current injection (blue trace). A −120 pA step current injection caused membrane hyperpolarization (blue trace), which gradually increased to its maximum value (−130 mV, dot in Figure 4C) and then depolarized slowly (−96 mV, sag) despite continued current injection. Return of the membrane potential to baseline was associated with the occurrence of a spontaneous action potential. Gray traces show the membrane potential changes in response to a positive and negative current step injection in the presence of the I_h_ blocker ZD7288 (10 microM/L). ZD7288 suppressed spontaneous action potential discharge during depolarizing current injection, and reduced the sag potential (difference between peak membrane potential and endpulse potential). **D**) Plots of the peak vs. endpulse voltage as a function of the magnitude of the hyperpolarizing step current injection under control and following application of ZD7288. ZD7288 suppressed sag potentials in Ah-type neurons. Date are mean ±1 SD. *n* = 6 cells for each data point, *P<0.05 and **P<0.01 *vs*. control (Ctrl).

Electrical characteristics of Ah-type VGNs are displayed in Figure 3. Values for CV (8.13 m/s; Figure 3A) and maximal upstroke velocity (306.6 mV/msec; blue trace in Figure 3B) were intermediate between those of A− and C-type neurons, whereas threshold potential was very similar to A-type neurons, and duration and shape (repolarization ‘hump’) of the AP resembled those of C-type cells (Figure 3B). Examples of hyperpolarization-induced sag potentials and their relation to I_h_ are summarized in Figure 3C and D. Depolarizing current injection triggered repetitive action potential firing. A hyperpolarizing current injection caused a gradually increasing membrane hyperpolarization to a peak value (dot in Figure 3C), followed by spontaneous depolarization (voltage sag; Figure 3C) to a steady potential at the end of the current pulse. Application of ZD7288 suppressed both spontaneous action potential firing in response to a depolarizing current injection step and the hyperpolarization-evoked sag potential (triangle in Figure 3C). The magnitude of the sag potential, i.e., the difference between the peak and the steady-state voltage during a hyperpolarizing current step, became progressively larger as the amplitude of the current step increased (Figure 3D). ZD7288 suppressed sag potentials over the entire range of current pulses tested (Figure 3D). Further, increases in the step current magnitude were associated with a progressive shortening of the time to peak voltage as well as a fastening of the sag repolarization rate (see Figure S3).

### I_h_-mediated PEMH Supports Continuous Action Potential Firings Evoked by Vagal Stimulation

**Figure 4:**
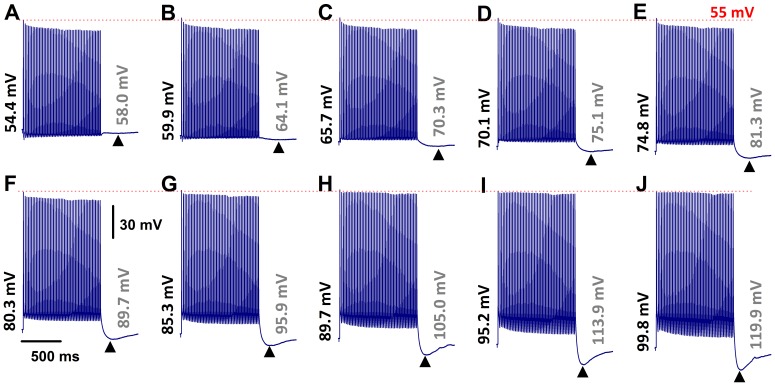
RMP hyperpolarization restores spike amplitude and accentuates post-excitatory membrane hyperpolarization in myelinated Ah-type VGNs from adult, non-ovariectomized rats. **A**) Train of vagal stimulation-evoked action potentials (50 Hz) in an Ah-type neuron under zero current-clamp condition. Note the decline in spike amplitude with increasing number of evoked action potentials, and a small-amplitude PEMH. **B**) – **J**) Effect of hyperpolarizing current injection on vagal stimulation-evoked action potentials. Numbers at the beginning and end of each trace denote the initial RMP and the minimal membrane potential during a PEMH, respectively. Hyperpolarizing currents were applied throughout the recordings, including the post-tetanic period. Vagal stimulation frequency was 50 Hz. Trains of action potentials during vagal stimulation Scale bars in (**F**) apply to all panels.

We also examined the effect of resting membrane hyperpolarization on the properties of rapid vagal stimulation-induced action potentials and PEMHs in Ah-type cells. Representative examples are shown in Figure 4. Under zero current-clamp conditions (Figure 4B), a 1-s train of vagal stimulation at 50 Hz caused a progressive decrease in the peak amplitude of evoked action potentials and a small-amplitude PEMH. The mean ±1 SD of the peak amplitude of the last evoked action potential in a train of 50 Hz was 79.2% ±4.8% (*n* = 11 cells from 8 slice preparations) of control. The average PEMH amplitude (defined as the difference between the pre-train RMP and the most negative potential during the PEMH) was −3.3±0.24 mV. The same stimulation protocol was then repeatedly applied to the same neuron during simultaneous hyperpolarizing current injections of progressively increasing amplitude, as demonstrated in Figure 4B–J. This maneuver evoked several distinct effects on the membrane potential of Ah-type neurons. First, increasing degrees of RMP hyperpolarization progressively enhanced peak PEMH amplitude (Figure S4A). Second, the difference between pre-train and end-train RMP also increased with increasing RMP hyperpolarization (Figure S4B), i.e., the same rate of rapid vagal stimulation caused progressively larger depolarizing shifts in RMP with increasing pre-train hyperpolarization. This gradual increase in RMP shift was associated with an increase in peak PEMH amplitude. Third, repetitive action potentials evoked from holding potentials ≤−80 mV shifted the peak of the PEMHs to progressively more negative values. For example, the peak amplitude of the PEMH in a train of 50 Hz was −89.7 mV at a RMP of −80.3 mV (Figure 4F) compared with −119.9 mV at a RMP of −99.8 mV (Figure 4J). The time constants of voltage relaxation during PEMHs progressively decreased with increasing hyperpolarization, being 375 ms at −70 mV, 306 ms at −80 mV, 239 ms at −90 mV, and 115 ms at −100 mV (Figure 4D, F, H and J). Concomitantly, a progressive decrease in peak amplitude of evoked action potentials was no longer observed at RMPs more negative than −85 mV (Figure 4H–J).

**Figure 5:**
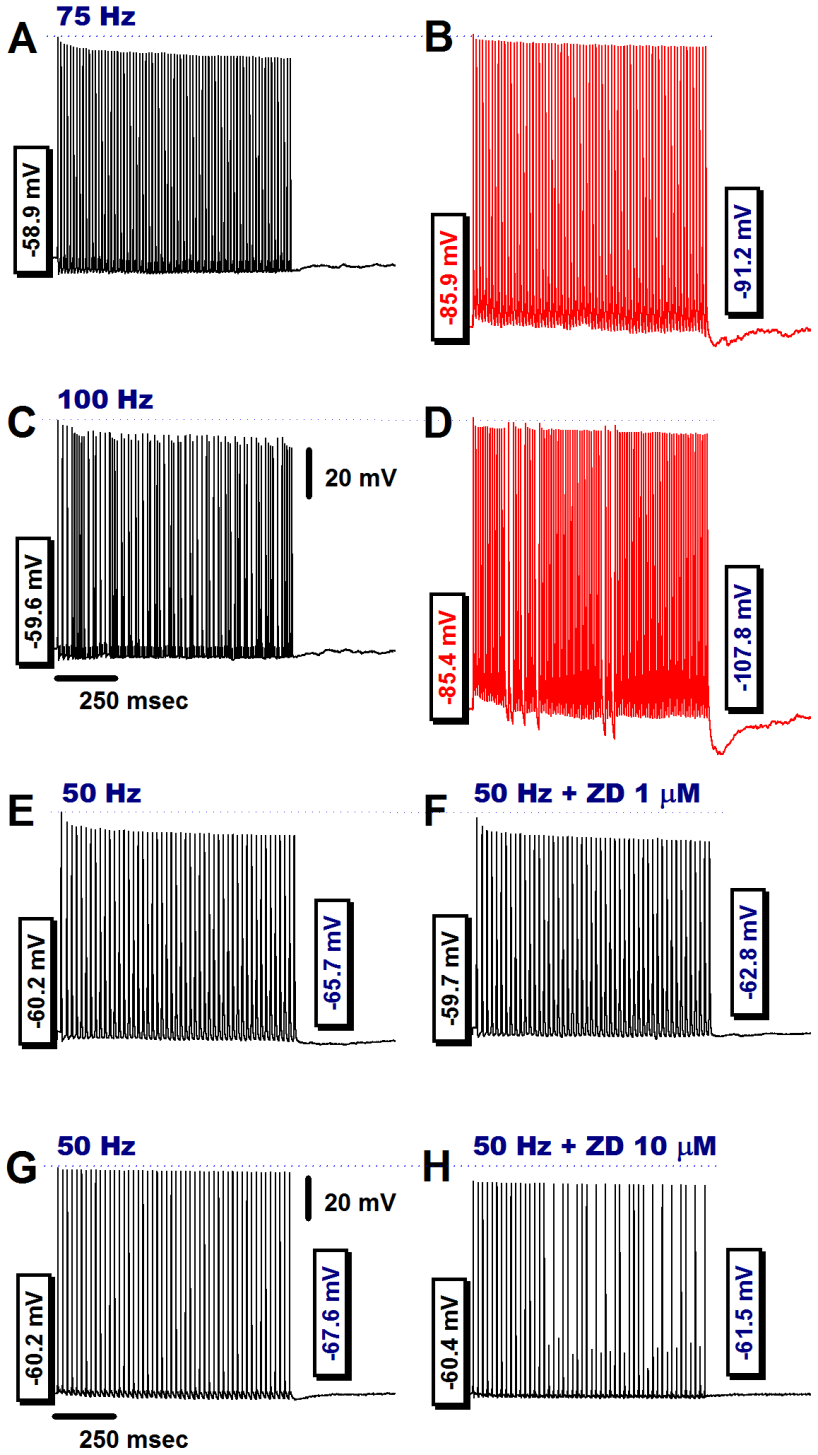
Hyperpolarization increases, whereas ZD7288 reduces, both PEMH amplitude in and excitability of Ah-type VGNs. **A**) Transmembrane potentials recorded under zero current-clamp condition in an Ah-type neuron before, during and after application of a train of vagal stimuli at 75 Hz. A 1∶1 stimulus/response ratio was sustained throughout the vagal stimulation episode. The peak amplitude of the evoked action potentials progressively decreased to a new steady state value at the end of the pulse train. No PEMH was observed. **B**) Sustained application of a hyperpolarizing current throughout vagal stimulation attenuated the reduction in peak amplitude of evoked action potentials in a train of 75 Hz and induced a PEMH. Hyperpolarizing currents were applied throughout the recordings, including the post-tetanic period. **C**) Further increase in the vagal stimulation frequency failed to induce a stable 1∶1 stimulus/response pattern under zero current-clamp conditions, rather a 3∶2 cycle developed, i.e. every third stimulus did not trigger an action potential. No PEMH was induced. **D**) Injection of a hyperpolarizing current resulted in restoration of a 1∶1 stimulus/response pattern throughout most of the train duration with only brief, intermittent 2∶1 cycles, and induced a large PEMH. Hyperpolarizing currents were applied throughout the recordings, including the post-tetanic period. **E**) and **F**) In a separate Ah-type neuron, application of ZD7288 (1 microM) eliminated the PEMH. **G**) and **H**) In another Ah-type VGN, a 1-s train of vagal stimulation at 50 Hz evoked action potentials at a 1∶1 stimulus/response ratio and a small-amplitude PEMH. Application of 10 microM ZD7288 to the preparation resulted in loss of a stable 1∶1 stimulus/response pattern and also abolished the PEMH. Scale bars in (**C**) also apply for (**A**), (**B**), and (**D**); scale bars in (**G**) are also applied to (**E**), (**F**), and (**H**).

In some Ah-type VGNs, we were able to evoke a stable 1∶1 stimulus/response pattern up to a vagal stimulation frequency of 75 Hz, as demonstrated in Figure 5A. Application of a sustained hyperpolarizing current to these neurons attenuated the decline in peak amplitude of the evoked action potentials and gave rise to a large-amplitude PEMHs (Figure 5B). Further increasing the frequency of vagal stimulation to 100 Hz was accompanied by a loss of the 1∶1 stimulus/response pattern, which was partially restored upon application of a hyperpolarizing current, concomitant with reappearance of a large-amplitude PEMH (Figure 5D).

To further test the potential role of I_h_ in contributing to the PEMH and in maintaining peak voltage of stimulated action potentials, we applied the I_h_ blocker ZD7288. The results, demonstrated in Figures 5E–H, suggest a role of I_h_ in both mediating PEMH and maintaining peak action potential amplitude. The latter finding is compatible with the notion that I_h_ provides direct depolarizing effect increasing peak action potential amplitude [28]. On average, application of 1 microM ZD7288 to Ah-type neurons did not significantly alter RMP (control: −60.4±4.8 mV, ZD7288: −60.7±4.3 mV; n = 6; *P*>0.05 by paired *t*-test) but significantly reduced peak PEMH amplitude (control: −5.98±1.15 mV, ZD7288: −2.41±0.67 mV; *P*<0.01 by paired *t*-test).

### I_h_ Recorded from Visceral Ganglion Neurons in Intact Ganglion Slice Preparations from Adult, Non-ovariectomized Rats

**Figure 6:**
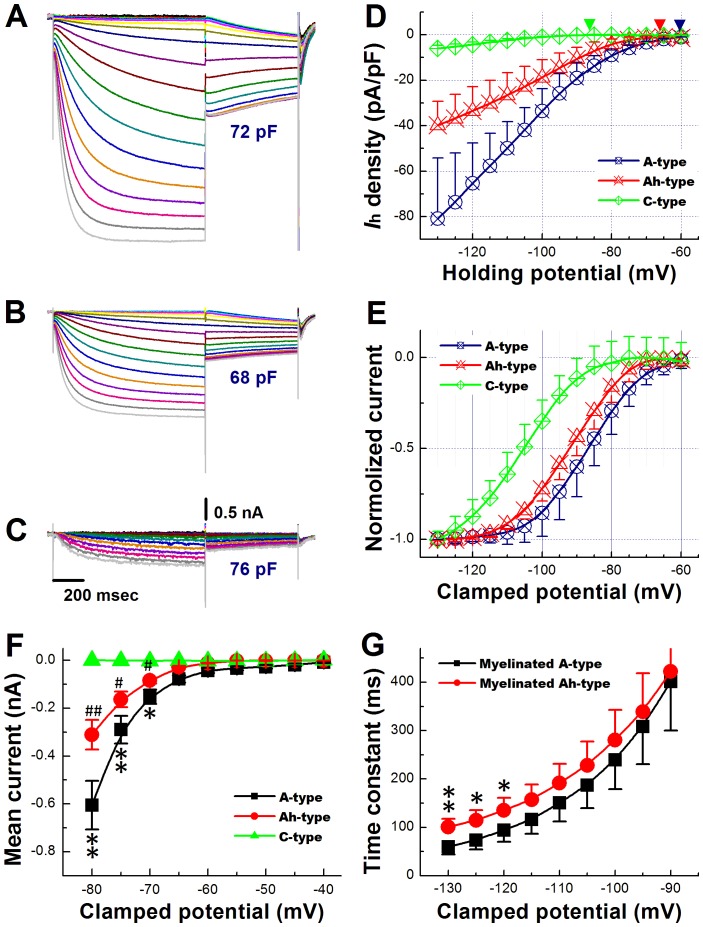
Characterization of I_h_ in VGNs from adult, non-ovariectomized rats. **A**), **B**), and **C**) Hyperpolarization-evoked currents recorded from an A−, Ah− and C-type neuron (panel A, B and C, respectively). Cells were subjected to a twin-pulse protocol, wherein they were held between −40 and −130 mV in steps of 5 mV for 1 second, and then clamped to −80 mV for 600 ms before return to the holding potential of −40 mV. The interval between each twin-pulse was 1 s. In the A− and Ah-type neuron, step hyperpolarizations evoked inward currents that had an instantaneous component followed by a slowly activating component, whereas the initial instantaneous component was not observed in the C-type neuron. The magnitude of hyperpolarization-evoked currents was largest in the A-type cell, intermediate in the Ah-type cell, and smallest in the C-type cell. Clamping the cell to −80 mV caused slowly activating or slowly deactivating tail currents, depending on the voltage of the preceding clamp step. **D**), Plots of hyperpolarization-evoked current densities as a function of voltage for all three neuronal subtypes. Endpulse currents were used for analyses. Data are mean ±1 SD. **E**) Activation curves obtained from tail current recordings as shown in (**A**), (**B**) and (**C**). Sigmoid fittings indicated half maximal activation at −88.0±3.14 mV, −92.4±4.5 mV, and −106±12.8 mV (*P*<0.01 by ANOVA) in A-, Ah−, and C-type cells, respectively (denoted by the downward arrows). Slopes of the activation curves were S_1/2_ = 6.14±1.22 mV, 6.13±0.98 mV, and 7.14±2.2 mV, *P*>0.05 by ANOVA ). **F**), Plots of inward current densities elicited during hyperpolarizations to potentials ranging from −40 to −80 mV. Scale bars in (**C**) also apply for (**A**) and (**B**). Data are mean ±1 SD, *P<0.05 and **P<0.01 vs Ah-type, ^#^ *P*<0.05 and ^##^ *P*<0.01 vs C-type. **G**) Time constants of Ih activation as a function of voltage in A and Ah type neurons. Time constants were obtained by monoexponential fit of the current traces in (**A**) and (**B**). Values are mean ± SD. *P<0.05 and **P<0.01 vs A-type.

Our current-clamp data strongly suggest that both A− and Ah-type VGNs express a ZD7288-sensitive current with properties typical of hyperpolarization-activated cyclic nucleotide gated (HCN) channels. To compare the density and biophysical properties of I_h_ among the three VGN subtypes, we performed whole-cell voltage-clamp recordings in slices from adult, non-ovariectomized rats. VGN subtypes were identified based on CV measurements. Examples of I_h_ currents evoked by a double-pulse voltage-clamp protocol are shown in Figure 6. The recordings revealed activation of a current at negative potentials (see Figure 6A, B and C) that gradually increased over time to reach steady state. Density of these hyperpolarization-evoked currents (measured at the end of a 1-s voltage step to −120 mV) was largest in A-type cells, intermediate in Ah-type cells, and smallest in C-type cells (Figure 6D). I_h_ activation curves obtained from tail currents are shown in Figure 6E. The half activation voltage, V_1/2_, was least negative in A-type neurons (−88.0±3.14 mV), intermediate in Ah-type neurons (−92.4±4.50 mV) and most negative in C-type cells (−106±12.8 mV; *P*<0.01 by ANOVA; see Figure 6E). Values for the slopes, S, of the activation curves were indistinguishable among the three subtypes (S_1/2_ = 6.14±1.22 mV, 6.13±0.98 mV, and 7.14±2.20 mV for A−, Ah−, and C-type cells, respectively; *P*>0.05 by ANOVA). In C-type VGNs, I_h_ density was small and currents activated more slowly and at more negative potentials compared with A− and Ah-type neurons (Figure 6C, D & E). Densities and biophysical properties of hyperpolarization-evoked currents are summarized in Table 1.

**Table 1:** Classification of afferent VGN subtypes and characterization of hyperpolarization-activated current (I_h_).

	Non-ovariectomized (Non-OVX)			Ovariectomized (OVX)		
Afferent	A-type	Ah-type	C-type	A-type	Ah-type	C-type
**(# of cells)**	(11)	(10)	(23)	(6)	(7)	(12)
**(# of animals)**	(5)	(6)	(7)	(4)	(4)	(6)
**CV (m/s)**	16.1±2.57	10.8±4.82*	0.59±0.02^‡^	15.7±3.35	11.5±4.69*	0.62±0.02^‡^
**WCC (pF)**	78.4±12.3	72.6±8.4	74.9±11.2	81.5±9.96	74.3±7.91	77.0±9.32
**I_h_ (pA/pF)**	−65.2±17.6	−27.9±10.7*	−4.33±1.06	−63.7±25.4	−12.1±4.13**^††^	−5.31±0.46^‡^
**V_1/2_ (mV)**	−88.0±3.14	−92.4±4.50	−106±12.8^‡^	−89.6±3.28	−101±5.31*^†^	108±15.9^‡^
**S_1/2_ (mV)**	6.04±1.22	6.13±0.98	7.14±2.20	6.10±1.34	6.16±1.52	7.10±2.05
**Tau (msec)**	62.5±11.2	94.7±8.41**	351±36.3^‡‡^	65.7±14.5	129±13.1**	361±57.7^‡‡^

CV: fiber conduction velocity; WCC: whole-cell capacitance; I_h_: current density measured at the end of a 1-s voltage step to −120 mV; V_1/2_ and S_1/2_: half-activation voltage and slope, respectively, of the activation curve; Tau: activation time constant at −120 mV. Data are mean ±1 SD, *P<0.05 and **P<0.01 *vs* A-type, ^‡^ *P*<0.05 and ^‡‡^ *P*<0.01 *vs* Ah− type, and ^†^ *P*<0.05 and ^††^ *P*<0.01 *vs* Non-OVX.

### Stimulus/Response Ratios in Ah-type VGNs from Adult, Ovariectomized Rats

**Figure 7:**
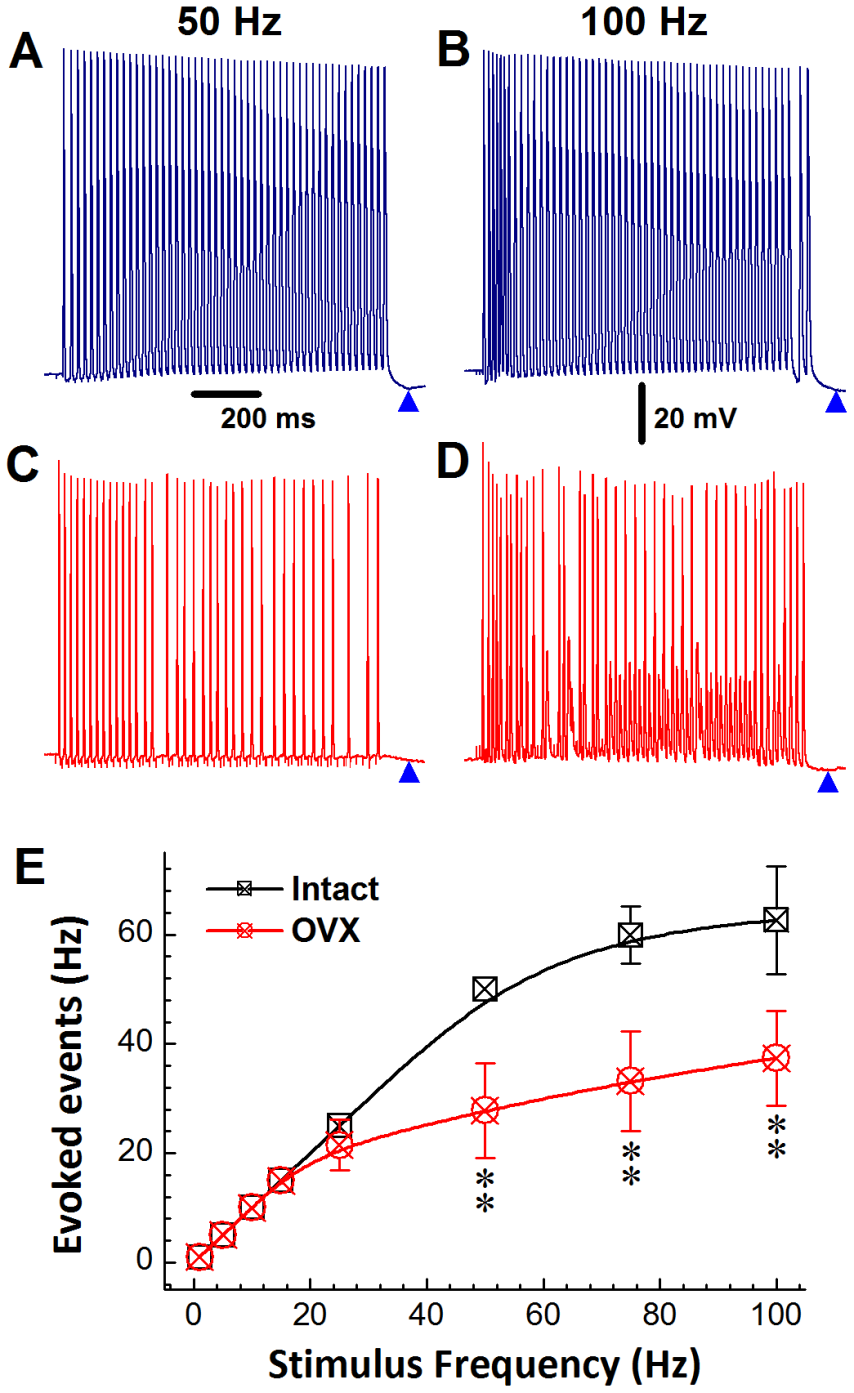
Electrical remodeling of Ah-type neurons in adult rats following ovariectomy. **A**) Train of somatic action potentials recorded during vagal stimulation at 50 Hz in an Ah-type neuron from a non-ovariectomized rat. The cell responded in a stable 1∶1 fashion to vagal stimulation. There was a progressive decline in the peak amplitude of the evoked action potentials in the train of 50 Hz, concomitant with a positive shift of the take-off potential. Arrowheads denote PEMHs. **B**) Doubling the vagal stimulation rate was no longer associated with a 1∶1 stimulus/response pattern of the same cell used in (A). **C**) and **D**), an Ah-type neuron from an ovariectomized rat failed to respond to vagal stimulation in a 1∶1 ratio at stimulation frequencies ≥50 Hz. **E**) Plots of incidence of evoked somatic action potentials as a function of vagal stimulation frequency in Ah-type VGNs isolated from non-ovariectomized and ovariectomized rats. Data are mean ±1 SD (*n* = 6) with **P<0.01 *vs* non-ovariectomized. Scale bar in (A) & (B) apply to all panels.

Ah-type VGNs from non-ovariectomized rats were able to sustain a 1∶1 stimulus/response ratio during vagal stimulation at frequencies ≤50 Hz (Figure 7A and B). Also, trains of rapid vagal stimulation readily evoked PEMHs (arrowheads in Figure 7A and B). In contrast, the maximal vagal stimulation frequency that still induced a stable 1∶1 stimulus/response pattern in Ah-type VGNs from ovariectomized rats was reduced, as shown in Figure 7C and D. No PEMHs were detectable in Ah-type neurons following cessation of rapid vagal stimulation. Plots of the incidence of vagal stimulation-evoked somatic action potentials in Ah-type VGNs as a function of stimulus frequency confirmed that cells from ovariectomized animals lost their 1∶1 stimulus/response pattern at significantly lower stimulation frequencies (>20 Hz) compared with non-ovariectomized animals (Figure 7E; *P*<0.01), but CV remained unchanged (see Table 1), suggesting reduced somatic excitability. These data suggested that HCN channels underlying I_h_ might be down-regulated and/or their properties altered following OVX.

### I_h_ Remodeling in Ah-type Visceral Ganglion Neurons from Ovariectomized Rats

**Figure 8:**
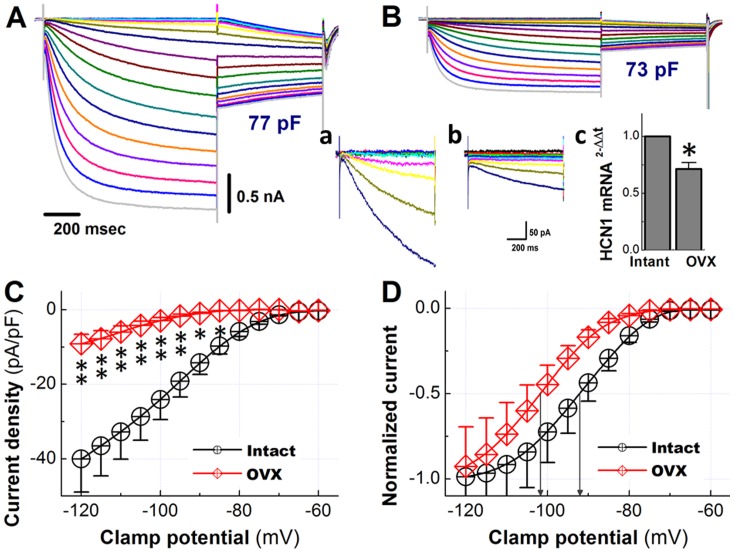
Characterization of the hyperpolarization-activated current in Ah-type vagal ganglion neurons from adult ovariectomized rats. **A**) and **B**), whole-cell current recordings in Ah-type neurons from a non-ovariectomized (**A**) and an ovariectomized rat (**B**). The same double-pulse voltage-clamp protocol as in Figure 7 was used. *Insert* **a**) and **b**), Highlight the endpulse currents at voltages from −45 mV to −80 mV at expanded y-scales, the scale bar in (**b**) also applies to (**a**). *Insert* **c**), Bar graph compares means of HCN1 mRNA levels in nodose ganglia from non-ovariectomized and ovariectomized rats. Data are from 6 rats per group. *P<0.05 by t-test. (**C**), Density of hyperpolarization-evoked currents as a function of clamp potential. *P<0.05 and **P<0.01 *vs* non-OVX. **D**), Activation curves of the tail currents obtained from recordings as in A and B. Sigmoid fittings indicated half maximal activation at −101±5.31 mV and −92.4±4.50 mV in OVX and non-OVX Ah-type neurons, respectively (denoted by the downward arrows; *P<*0.05). Slopes of the activation curves were S = 6.13±0.98 mV (non-OVX) and 6.16±1.52 mV (OVX; *P*>0.05). Scale bars in (**A**) also apply to (**B**).

Hyperpolarization-activated current has long been known to critically regulate excitability of various neuronal cell types, but its function in Ah-type VGNs has not been previously addressed. Accordingly, we performed voltage-clamp recordings in Ah-type cells in nodose ganglion slices from non-OVX and OVX rats. Representative results are demonstrated in Figure 8A and B. These recordings revealed activation of currents at negative potentials, which gradually increased over time to reach a steady-state at the end of 1-s clamp pulses to potentials ranging from −40 to −130 mV. Stepping to a test potential of −80 mV followed by the 1-s pre-pulse evoked slowly activating or slowly deactivating tail currents (Figure 8A). The density of the hyperpolarizing current (measured at the end of 1-s long test potentials to −120 mV) was significantly reduced in Ah-type cells from ovariectomized rats (Figure 8C). OVX induced a negative shift of the activation curve (non-OVX: −92.4±4.50 mV, OVX: −101±5.31 mV; *P*<0.05), but did not alter its steepness factor S (non-OVX: 6.13±0.98 mV, OVX: 6.16±1.52 mV; *P*>0.05, Figure 8D). The down-regulation of I_h_ density was paralleled by a reduction of HCN1 mRNA levels in nodose ganglia from OVX rats (Figure 8F).

### Effects of 17beta-estradiol on I_h_ Density in Ah-type VGNs from Ovariectomized Rats

**Figure 9:**
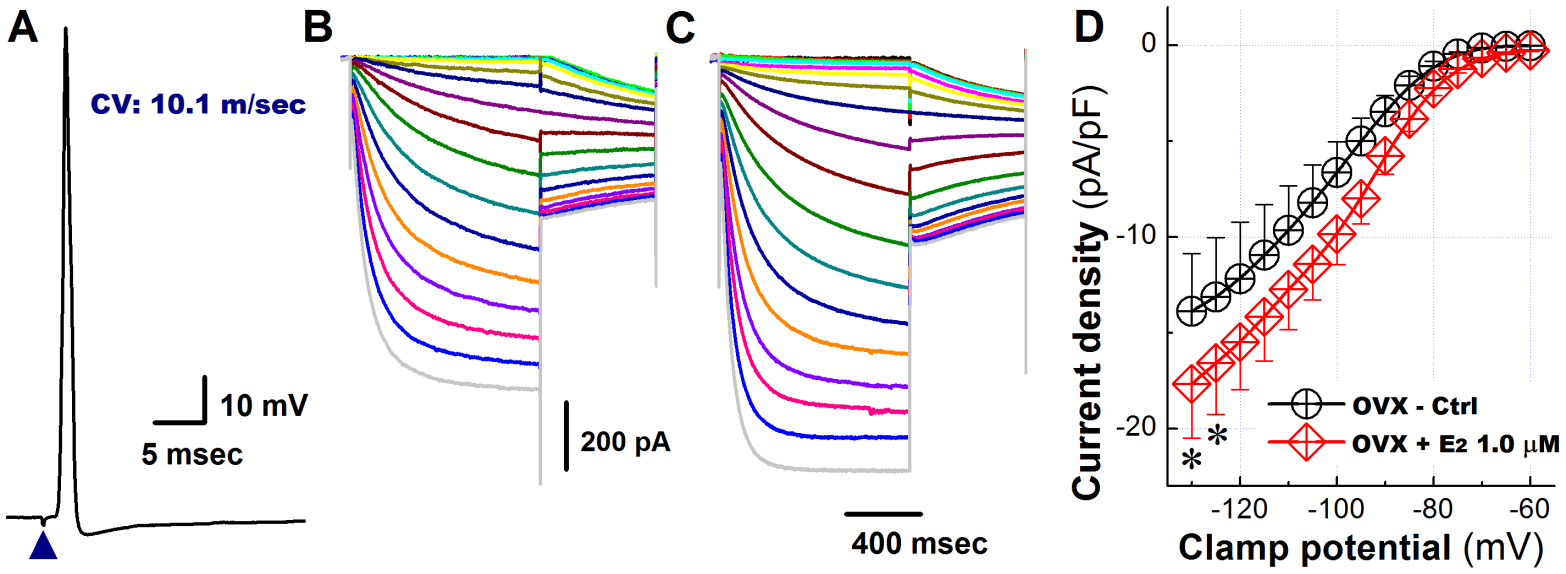
17beta-estradiol partially restores the hyperpolarization-activated currents in Ah-type vagal ganglion neurons from adult ovariectomized female rats. **A**) Somatic action potential recorded from an Ah-type neuron in response to a single vagal nerve stimulus (arrowhead). The CV was found to be 10.1 m/s and no repolarization ‘hump’ was detectable, indicative of an Ah-type cell. **B**) and **C**), Families of hyperpolarization-evoked currents recorded in an Ah-type neuron from an OVX animal before (**B**) and following application of 1 microM/L 17beta-estradiol (**C**) to the bath solution. **D**) Plots of hyperpolarizing current density versus clamp potential in the absence and presence of 1 microM/L 17beta-estradiol. Data are mean ±1 SD (*n* = 6) with *P<0.05 and **P<0.01 *vs* OVX Ctrl.

We previously demonstrated that 17beta-estradiol (17beta-E_2_) acutely restores neuronal excitability of Ah-type VGN from OVX rats [10]. The results presented here demonstrate that hyperpolarization-evoked currents recorded from Ah-type VGNs are significantly reduced in nodose ganglia from OVX rats (Figure 8A–C) and, further, their activation curve is shifted to more negative potentials (Figure 8D). To examine the possibility that changes in I_h_ density and properties directly result from estrogen deficiency, we measured I_h_ both in the absence and presence of 1 microM/L 17beta-estradiol (17beta-E_2_). The results are summarized in Figure 9. In fact, 17beta-E_2_ was able to significantly increase I_h_, at least at very negative clamp potentials (Figure 9B – D, and Table 2) and also to significantly accelerate I_h_ activation (Table 2). Overall, these results are compatible with the notion that ovariectomy-induced I_h_ remodeling, at least in part, directly results from estrogen deficiency.

**Table 2:** Effect of 1.0 microM 17beta-estradiol (17beta-E_2_) on I_h_ in myelinated Ah-type VGNs from ovariectomized (OVX) rats.

Ah-type afferents from an OVX female		
Profile	Control	1 microM 17beta-E_2_
**I_h_ (pA/pF)**	14.07±3.01	17.65±2.81*
**V_1/2_ (mV)**	−103.2±7.02	−99.9±5.58
**S_1/2_ (mV)**	6.46±2.37	6.05±1.98
**Tau (msec)**	136±12.16	107±9.63*

See Table 1 for explanation of V_1/2_, S_1/2_, and activation time constant (Tau). Data are mean ±1 SD with *P<0.05 *vs* control, *n* = 5 VGN from 5 ganglion preparations.

## Discussion

### Major Findings

Using an established rat model of experimentally induced postmenopausal estrogen deficiency, we have shown that Ah-type vagal nodose ganglion neurons from ovariectomized adult rats exhibit a flattened stimulus-response curve, a ∼75% reduction in I_h_ density, a shift of the I_h_ activation curve to more negative potentials, and slowed I_h_ activation. Further, acute exposure of nodose ganglion slices from ovariectomized rats to 17beta-estradiol partially restored density and activation properties of I_h_ in Ah-type neurons. Finally, expression of mRNA encoding the isoform 1 of the hyperpolarization-activated cyclic nucleotide-gated (HCN1) channel was reduced by ∼30% in nodose ganglia from rats with surgical ovariectomy. Since ovariectomy did not significantly alter afferent CV of Ah-type neurons, our results support the notion that chronic estrogen deficiency leads to alterations in density and biophysical properties of I_h_ in Ah-type VGNs. Further, reduced I_h_ density in ovariectomized rats may result, at least partially, from diminished HCN1 gene transcription. Ovariectomy-induced electrophysiological remodeling of Ah-type VGNs may contribute to alterations in cardiovascular physiology typically observed in postmenopausal women. Specifically, a decreased activity in baroreceptor afferents would be expected to increase sympathetic outflow via the baroreceptor reflex. Thus, decreased excitability after ovariectomy might increase systemic blood pressure.

### Unique Advantages of the Nodose Ganglion Slice Preparation for the Study of VGN Electrophysiology

The intact nodose slice preparation that was used in this study provides two major advantages over the use of isolated neurons for the study of VGN electrophysiology. First, VGN subtypes can be reliably and unambiguously distinguished from each other based on their afferent CV. Second, the vagal stimulus-response pattern of individual VGNs can be measured. One ‘limitation’ of this preparation is the restricted diffusional access of deeper cell layers, requiring longer equilibration times for wash-in and -out of pharmacological agents.

Using afferent CV as a definite marker of neuronal subtypes, we were able to identify Ah-type cells as a target of ovariectomy-induced electrical remodeling. More specifically, surgical ovariectomy was associated with down-regulation of a hyperpolarization-activated inward current that exhibited the typical features of an HCN-encoded ion channel, i.e., sigmoidal dependence on activation at potentials negative to −40 mV, a slowly activating component of the hyperpolarization-induced inward current, and slow deactivation upon depolarization. Injections of negative currents caused a biphasic voltage response in Ah-type VGNs, consisting of an initial, gradually developing hyperpolarization followed by a slow depolarization (‘sag’) to a new steady state potential. Also, rebound action potentials occurred upon release of the current injection. The sag potential and rebound action potential were suppressed by the I_h_ blocker ZD7288, in agreement with our previous observations [13] and those by others [16], [17], [26]. These considerations indicate that Ah-type VGNs express functional, hyperpolarization-activated cyclic nucleotide-gated channels.

### I_h_ Contributes to Excitability of VGNs

In the present study, the I_h_ blocker ZD7288 was found to reduce the maximal frequency at which electrical vagal stimulation still caused a 1∶1 somatic response in Ah-type neurons (Figure 5), suggesting a role of HCN channels in modulating Ah cell excitability. Given the previous observation by others that HCN channels are not only expressed in the soma but also in dendrites/axons of other neuron types [29], we cannot exclude the possibility that the changes in the vagal stimulus-response pattern of Ah-type neurons in response to ZD7288 arise as a consequence of I_h_ inhibition along the afferent axons. Because the patch electrode records changes in transmembrane currents from impaled cells in their entirety, not only from the soma, the voltage-clamp measurements cannot help distinguish effects on the nerve versus soma. Future immunocytological studies and electrical recordings from the dendritic/axonal membranes using the loose-patch approach will be necessary to answer this question. We have to emphasize, however, that ovariectomy was not associated with a decrease in afferent CV of Ah-type neurons despite pronounced down-regulation of I_h_ density, suggesting that I_h_ does not contribute to axonal excitability and/or its downregulation is restricted to the soma.

Our findings are compatible with the previous observation by others that I_h_ provides a depolarizing, i.e., excitatory, current at subthreshold potentials, which increases the resting membrane conductance (that is, it lowers the input resistance), thereby regulating the excitability of VGNs. Besides from pharmacological I_h_ inhibition, the role of I_h_ in controlling VGN excitability is also inferred from the observation that C-type cells exhibit a very low I_h_ density and pronounced leftward shift in the I_h_ activation curve compared to both A− and Ah-type cells, which very well correlate with the diminished ability of C-class neurons to maintain a 1∶1 stimulus-response pattern at vagal stimulation frequencies exceeding 20 Hz (Figure S2). Further, ovariectomy induces a flattening of the stimulation-response curve in Ah-type neurons, which coincides with a profound reduction in I_h_ density as well as a hyperpolarizing shift in and a slowing of I_h_ activation. It is somewhat puzzling that the I_h_ blocker ZD7288 did not cause resting membrane hyperpolarization in our study. Several explanations are possible. First, I_h_ around the RMP is of low magnitude in vagal ganglion neurons (e.g., −12 pA at −60 mV for Ah type neurons; see Figure 6F). Such small-sized currents may be insufficient to measurably influence RMP, in particular when the resting input resistance is low. In this context it is noteworthy that we have previously found the average RMP of A-type neurons not to be significantly different from that of C-type neurons [25], despite their marked differences in I_h_ density and properties. Second, ZD7288 blockade of HCN channels has previously been shown to be relieved on hyperpolarization. For example, Shin and co-workers found a sigmoidal dependence of ZD7288 blockade of heterologeously expressed HCN1 channels on membrane voltage, with a voltage at half-maximal block of −117.5 mV and a steepness factor of 4.2 mV [30]. Assuming a similar voltage-dependence of ZD7228-induced HCN channel blockade in VGNs, one would expect effective channel block at the physiological resting membrane potential of these cells (between −70 to −60 mV; Li and Schild, J Neuroscience Methods). It therefore seems unlikely that the voltage-dependence of ZD7288 blockade of HCN channels explains the failure of the drug to noticeably alter RMP in our experiments, unless HCN channels in their native environment exhibit a markedly different voltage-sensitivity of ZD7288 inhibition compared to their ectopically expressed counterparts.

### Transient Deactivation of I_h_ Contributes to PEMHs in A− and Ah-type Neurons

Both A− and Ah-type fibers develop prolonged afterhyperpolarizations (PEMHs) in response to trains of vagal stimulation. The peak PEMH amplitude progressively increases with the cumulative number of spikes preceding the PEMH. Application of a hyperpolarizing current increased, whereas application of ZD7288 reduced peak amplitudes of PEMHs both in A− and Ah-type neurons under our experimental conditions. This observation is compatible with the notion that PEMHs evoked by repetitive action potentials were mediated, at least in part, by transient deactivation of HCN channels that underlie inward I_h_. The PEMH amplitudes in the absence of a hyperpolarizing current were in the range from −4 to −8 mV. Assuming an input resistance of 350 Mega-ohm for nodose ganglion neurons (see above; [31]), a 22-pA change in net current can be predicted from Ohm’s law to give rise to a PEMH amplitude of 8 mV. Mean I_h_ amplitudes measured at the end of 1-s voltage steps to −60 mV and −65 mV (i.e., the RMP in A− and Ah-type neurons [see Figures 6 and 8]) in the present study ranged from −12 pA to −28 pA in Ah-type neurons and from −42 pA to −77 pA in A-type neurons. Thus, the magnitudes of inward I_h_ are sufficiently large to contribute a 4 - 8-mV depolarizing influence on the resting membrane potential. We also found that for the same vagal stimulation rate the magnitude of the depolarizing shift during the spike train gradually increased with increasing pre-train membrane hyperpolarization, and that the peak PEMH amplitude rose with increments in the depolarizing shift (Figure S4B). This observation is compatible with the notion that I_h_ deactivation requires sustained membrane depolarization, and that I_h_ is deactivated more the more negative the clamp potential, resulting in larger PEMH amplitudes. Further, we found that the time constant of voltage relaxation during PEMHs decreased with increasing membrane hyperpolarization (see Figure 4). This behavior is paralleled by a gradual decrease in the I_h_ activation time constant with increasing hyperpolarization as illustrated in Figure 6G, supporting the notion that I_h_ re-activation underlies the return of the membrane potential to pre-train values. Finally, this progressive acceleration of the rate of return to pre-train voltages with increasing hyperpolarization is mirrored by a similar kinetics behavior of the sag potentials elicited by hyperpolarizing current steps (see Figure S3), further strengthening the role of I_h_ re-activation in PEMHs.

A role of transient deactivation of I_h_ in contributing to post-tetanic hyperpolarization has previously been identified in striatal cholinergic interneurons [32], hippocampal neurons of wild type mice and of littermates lacking the alpha-5-subunit of the gamma-aminobutyric acid type A (GABA_A_) receptor [33], and in hippocampal neurons of rats [34]. Intriguingly, the latter study found a decrease in repetitive stimulation-induced, ZD7288-sensitive membrane hyperpolarization in the GABA_A_ receptor mutant mice concomitant with the decrease in I_h_ density.

The observation that the I_h_ inhibitor ZD7288 significantly reduced PEMHs, but had no discernible effects on RMP of nodose ganglion neurons, is puzzling. We propose two mechanisms that may explain this finding. The first mechanism involves dynamic regulation of resting input resistance in VGNs, i.e., the resting input conductance transiently decreases following repetitive vagal stimulation, causing the same size of I_h_ to give rise to a larger depolarizing influence. Interestingly, such dependence of neuronal excitability on electrical activity has previously been described in myenteric Ah neurons [35] and in murine hippocampal neurons [33]. Second, it is possible that repetitive spike activity modulates the activation properties of HCN channels, resulting in a depolarizing shift and/or increased steepness of the I_h_ activation curve. Indeed, an activity-dependent increase in cytosolic cAMP has previously been shown to result in transiently enhanced I_h_ in neurons secondary to a positive shift of the channel’s activation curve [36]. Similarly, repetitive stimulation has been shown to upregulate HCN channels in rat hippocampal neurons [34]. Future investigation will have to clarify the mechanisms underlying the apparent discrepancy of ZD7288 effects at rest and following repetitive electrical activity of VGNs.

### It is Possible that ZD7288 Modulates the Activity of other Ion Channel/Transporters that Underlie PEMHs

For example, Nakajima et al. and Parker et al. previously provided evidence for a role of a Na-K electrogenic pump in contributing to post-tetanic hyperpolarizations in non-mammalian neurons [37], [38]. Thus, the trains of action potentials could have lead to intracellular Na^+^ accumulation in A− or Ah-type neurons which in turn activated a Na/K ATPase, causing transient hyperpolarization. However, it is noteworthy that application of ZD7288 at a concentration of 20 microM had no discernible effect on Na/K ATPase-mediated currents in cortical layer 5 neurons from rats [39], largely excluding the possibility that attenuation of PEMH amplitude resulted from drug-induced block of the electrogenic pump under our experimental conditions.

Another candidate underlying PEMHs is the large-conductance, Ca^2+^-activated K^+^ (BK) channel. Although cells were dialyzed with the Ca^2+^-chelator BAPTA, the possibility remains that sub-plasmalemmal increase in cytosolic Ca^2+^ levels occurred in response to high frequency vagal nerve stimulation, leading to temporary activation of BK channels and associated post-tetanic membrane hyperpolarization. However, the observation that the peak of the PEMH continues to become increasingly more hyperpolarized even at voltages more negative than the K^+^ equilibrium potential (E_K_) under our ionic conditions (−85 mV) is incompatible with the activity of BK channels as the only mechanism underlying PEMHs in A− and Ah-type neurons. We cannot rule out the possibility that K^+^ efflux through activated BK channels contributes to PEMHs at potentials less negative than E_K_, although the presence of a high-affinity Ca^2+^ buffer would be expected to lessen the likelihood of this occurring. The latter possibility would also imply that BK channels are inhibited by ZD7288, either directly or indirectly, which has not been documented previously.

### Ovariectomy Induces I_h_ Remodeling in Ah-type VGNs

Ovariectomy flattened the stimulus-response curve of Ah-neurons, indicating reduced excitability. It also markedly decreased I_h_ density and shifted the I_h_ activation curve leftward. These findings support the hypothesis that alterations in I_h_ density and biophysical properties act synergistically to significantly reduce excitability of Ah-type neurons. It is noteworthy that ovariectomy converts the high-frequency stimulus - response pattern of Ah-type neurons to a more C-type – like pattern, which in turn correlates very well with the markedly reduced I_h_ density and leftward shift of the I_h_ activation curve in these cells. We have not determined, however, whether alterations in I_h_ occur at the axonal or somatic level, or both. Interestingly, however, the stimulus/response pattern of Ah-type neurons from ovariectomized rats resembled that of A-type neurons in the presence of ZD7288, further strengthening the causation between I_h_ remodeling and altered Ah-cell excitability. On the other hand, I_h_ densities in Ah-type neurons from ovariectomized rats were not significantly different from those in neurons from non-ovariectomized rats at physiological membrane potentials (see Figure 9 C), arguing against a role of I_h_ remodeling in effecting changes in Ah-type excitability in estrogen-deficient animals. We have to emphasize, however, that the amplitudes of I_h_ at test potentials ranging from −55 mV to −80 mV, both in the OVX and in non-OVX cohort, were very small, making statistical comparisons challenging. In the examples shown in the insets of Figures 9 A and B, endpulse I_h_ amplitudes at test potentials ranging from −45 mV to −70 mV were larger in the non-OVX cell compared to the OVX cell. Finally, the observations that downregulation of HCN1 transcription paralleled decreases in I_h_ density and acute exposure to a synthetic estrogen partially restored I_h_ density in Ah-type neurons from OVX rats lend further support to a concept wherein HCN channels constitute a key target mediating changes in cardiovascular regulation seen in postmenopausal women.

The up-regulation of I_h_ density in Ah-type neurons from OVX rats upon short-term exposure to exogenous 17beta-estradiol is unlikely to result from a change in HCN gene transcription but rather suggests a post-translational modification of the HCN protein as an underlying mechanism. A number of previous studies by others have demonstrated that exogenous 17beta-estradiol at sub-micromolar concentrations can acutely alter neuronal electrophysiology via interaction with extranuclear estrogen receptors [40]. For example, the Kelly laboratory identified a G-protein coupled plasma membrane estrogen receptor in hypothalamic neurons which could be activated by 17beta-estradiol, leading to modulation of K^+^ currents via the phospholipase C – PKC/PKA pathway. The second messenger pathway involved in acute up-regulation of I_h_ in Ah-type neurons from OVX rats is unclear [41]. However, its identification in future studies may help establish a novel therapeutic target.

## Supporting Information

Figure S1 **Transmembrane voltage recording during the same stimulus train shown in** **Figure 1H** **at an expanded time scale.** Note the gradual increase in the peak amplitudes of the afterhyperpolarizations following each stimulated action potential during the train.
(DOCX)

Figure S2 **Electrical properties of unmyelinated C-type vagal ganglion neurons in the nodose ganglion preparation of adult non-ovariectomized rats.** **A**) Vagal stimulation-evoked transmembrane action potential in an unmyelinated C-type vagal ganglion neuron (VGN). The value for the conduction velocity (CV) measured between the stimulation and recording site was indicative of a C-type cell. **B**) Transmembrane action potential recorded from the same neuron as in (A) and its first derivative over time (blue trace). **C**) Transmembrane potentials recorded from an A-type neuron before, during and following 1-s vagal stimulation at 20 Hz. No PEMH was induced. **D**) Vagal stimulation at 35 Hz was associated with a variable action potential response pattern of C-type neurons. Scale bars in (**C**) also apply to all panels.
(DOCX)

Figure S3 **Hyperpolarization-evoked sag potentials in an Ah type neuron.** Graph shows voltage responses to hyperpolarizing current injections of increasing magnitude. Cell was held at −60 mV. The cell was injected with a maximum of −120 pA decreasing by −20 pA for every sweep. Numbers next to arrowheads indicate time to peak hyperpolarization; tau indicates the time constant of the ensuing depolarizing change in membrane potential. Tau values were obtained by mono-exponential fits of the data between the peak and the end-pulse voltages. Note the appearance of spontaneous action potentials upon relaxation of the hyperpolarizing current step.
(DOC)

Figure S4 **The relationship of deltaPEMH and clamped-potential (4A: top panel) or resting membrane potential (deltaRMP, 4B: bottom panel) 4A: X-axis: clamp potential; Y-axis: difference between the peak PEMH and the clamp potential (deltaPEMH).** **4B: X-axis**: difference between RMP and clamp potential (deltaRMP); **Y-axis**: deltaPEMH. Average data were presented as mean ±1SD, *n* = 5 complete sets of recordings.
(DOCX)

Table S1 **Electrical properties of myelinated Ah-type neurons in intact slices from control (no surgery), ovariectomized (OVX) and sham-operated adult rats.**
(DOCX)
