# Supplementary material for: Remodeling of Hyperpolarization-Activated Current, Ih, in Ah-Type Visceral Ganglion Neurons Following Ovariectomy in Adult Rats
Source: PLoS One. 2013 Aug 12;8(8):e71184. doi: 10.1371/journal.pone.0071184 (PMC3741359; doi:10.1371/journal.pone.0071184)

**Figure S1:** Transmembrane voltage recording during the same stimulus train shown in Figure 1H at an expanded time scale. Note the gradual increase in the peak amplitudes of the afterhyperpolarizations following each stimulated action potential during the train.


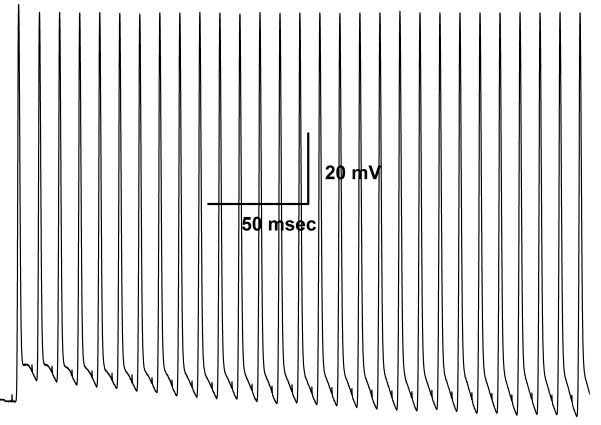

Supplement: Figure S1 — Transmembrane voltage recording during the same stimulus train shown in Figure 1H at an expanded time scale. Note the gradual increase in the peak amplitudes of the afterhyperpolarizations following each stimulated action potential during the train. (DOCX) [file pone.0071184.s001.docx]
