# Supplementary material for: Remodeling of Hyperpolarization-Activated Current, Ih, in Ah-Type Visceral Ganglion Neurons Following Ovariectomy in Adult Rats
Source: PLoS One. 2013 Aug 12;8(8):e71184. doi: 10.1371/journal.pone.0071184 (PMC3741359; doi:10.1371/journal.pone.0071184)
Supplement: Figure S2 — Electrical properties of unmyelinated C-type vagal ganglion neurons in the nodose ganglion preparation of adult non-ovariectomized rats. A) Vagal stimulation-evoked transmembrane action potential in an unmyelinated C-type vagal ganglion neuron (VGN). The value for the conduction velocity (CV) measured between the stimulation and recording site was indicative of a C-type cell. B) Transmembrane action potential recorded from the same neuron as in (A) and its first derivative over time (blue trace). C) Transmembrane potentials recorded from an A-type neuron before, during and following 1-s vagal stimulation at 20 Hz. No PEMH was induced. D) Vagal stimulation at 35 Hz was associated with a variable action potential response pattern of C-type neurons. Scale bars in (C) also apply to all panels. (DOCX) [file pone.0071184.s002.docx]

**Figure S2:** Electrical properties of unmyelinated C-type vagal ganglion neurons in the nodose ganglion preparation of adult non-ovariectomized rats. **A**) Vagal stimulation-evoked transmembrane action potential in an unmyelinated C-type vagal ganglion neuron (VGN). The value for the conduction velocity (CV) measured between the stimulation and recording site was indicative of a C-type cell. **B**) Transmembrane action potential recorded from the same neuron as in (A) and its first derivative over time (blue trace). **C**) Transmembrane potentials recorded from an A-type neuron before, during and following 1-s vagal stimulation at 20 Hz. No PEMH was induced. **D**) Vagal stimulation at 35 Hz was associated with a variable action potential response pattern of C-type neurons. Scale bars in (**C**) also apply to all panels.

**
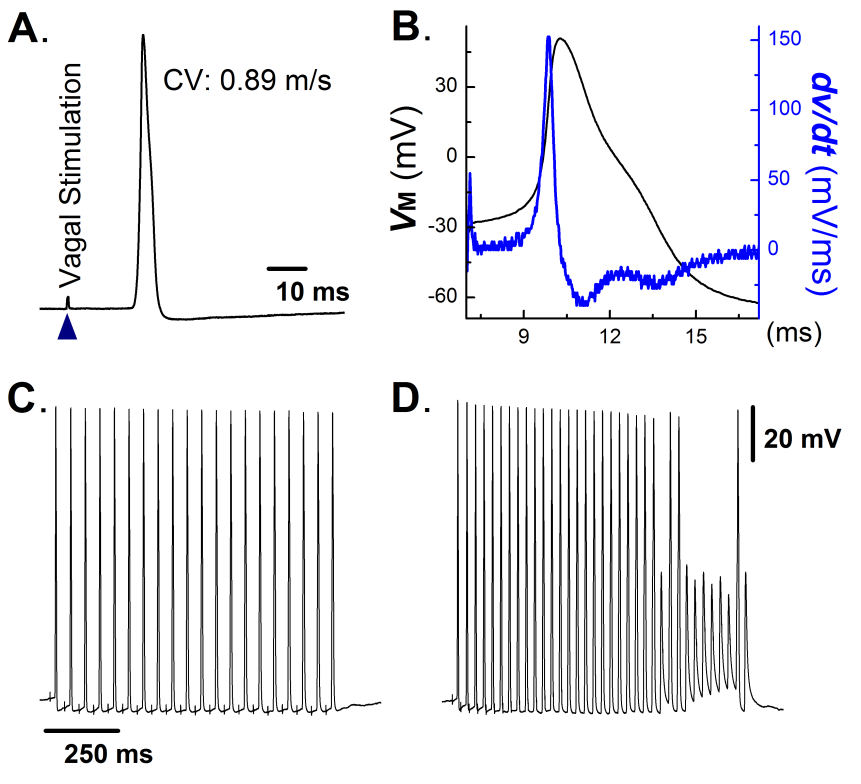
**
