# Supplementary material for: Remodeling of Hyperpolarization-Activated Current, Ih, in Ah-Type Visceral Ganglion Neurons Following Ovariectomy in Adult Rats
Source: PLoS One. 2013 Aug 12;8(8):e71184. doi: 10.1371/journal.pone.0071184 (PMC3741359; doi:10.1371/journal.pone.0071184)

**Figure S3:** Hyperpolarization-evoked sag potentials in an Ah type neuron. Graph shows voltage responses to hyperpolarizing current injections of increasing magnitude. Cell was held at -60 mV. The cell was injected with a maximum of -120 pA decreasing by -20 pA for every sweep. Numbers next to arrowheads indicate time to peak hyperpolarization; tau indicates the time constant of the ensuing depolarizing change in membrane potential. Tau values were obtained by mono-exponential fits of the data between the peak and the end-pulse voltages. Note the appearance of spontaneous action potentials upon relaxation of the hyperpolarizing current step.


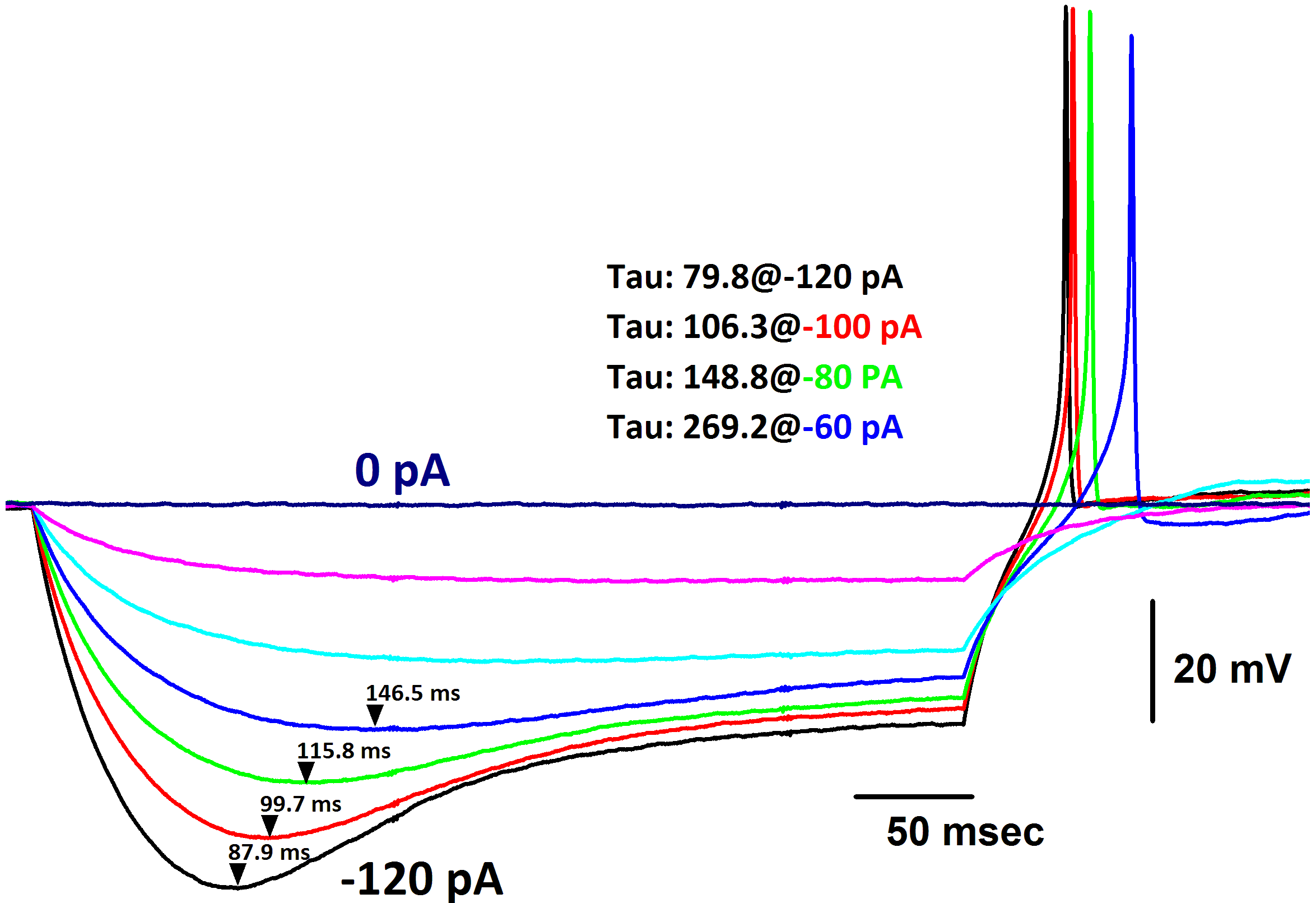

Supplement: Figure S3 — Hyperpolarization-evoked sag potentials in an Ah type neuron. Graph shows voltage responses to hyperpolarizing current injections of increasing magnitude. Cell was held at −60 mV. The cell was injected with a maximum of −120 pA decreasing by −20 pA for every sweep. Numbers next to arrowheads indicate time to peak hyperpolarization; tau indicates the time constant of the ensuing depolarizing change in membrane potential. Tau values were obtained by mono-exponential fits of the data between the peak and the end-pulse voltages. Note the appearance of spontaneous action potentials upon relaxation of the hyperpolarizing current step. (DOC) [file pone.0071184.s003.doc]
