# Supplementary material for: Remodeling of Hyperpolarization-Activated Current, Ih, in Ah-Type Visceral Ganglion Neurons Following Ovariectomy in Adult Rats
Source: PLoS One. 2013 Aug 12;8(8):e71184. doi: 10.1371/journal.pone.0071184 (PMC3741359; doi:10.1371/journal.pone.0071184)
Supplement: Figure S4 — The relationship of deltaPEMH and clamped-potential (4A: top panel) or resting membrane potential (deltaRMP, 4B: bottom panel) 4A: X-axis: clamp potential; Y-axis: difference between the peak PEMH and the clamp potential (deltaPEMH). 4B: X-axis: difference between RMP and clamp potential (deltaRMP); Y-axis: deltaPEMH. Average data were presented as mean ±1SD, n = 5 complete sets of recordings. (DOCX) [file pone.0071184.s004.docx]

**Figure S4: A** (upper panel) **and B** (bottom panel)


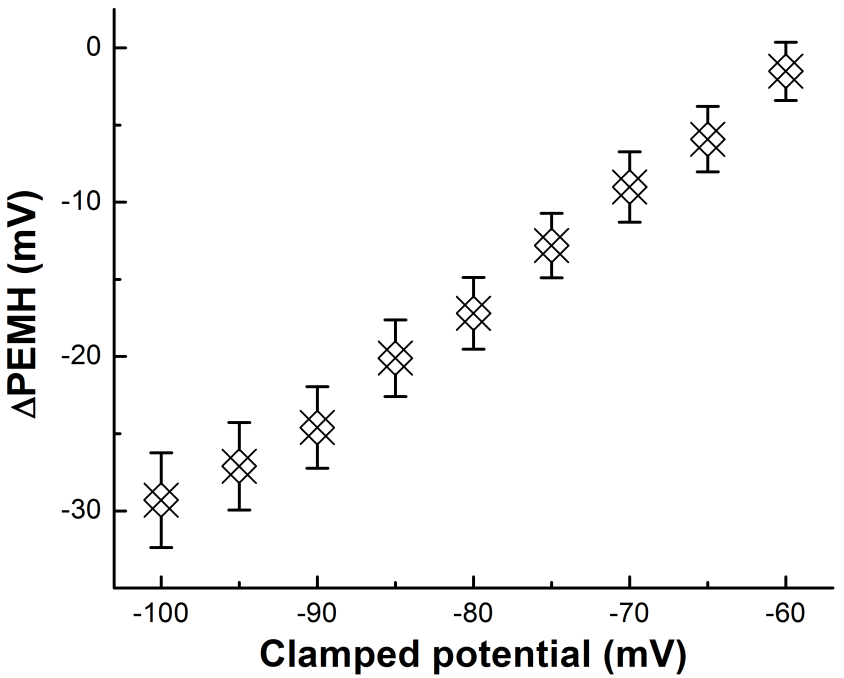


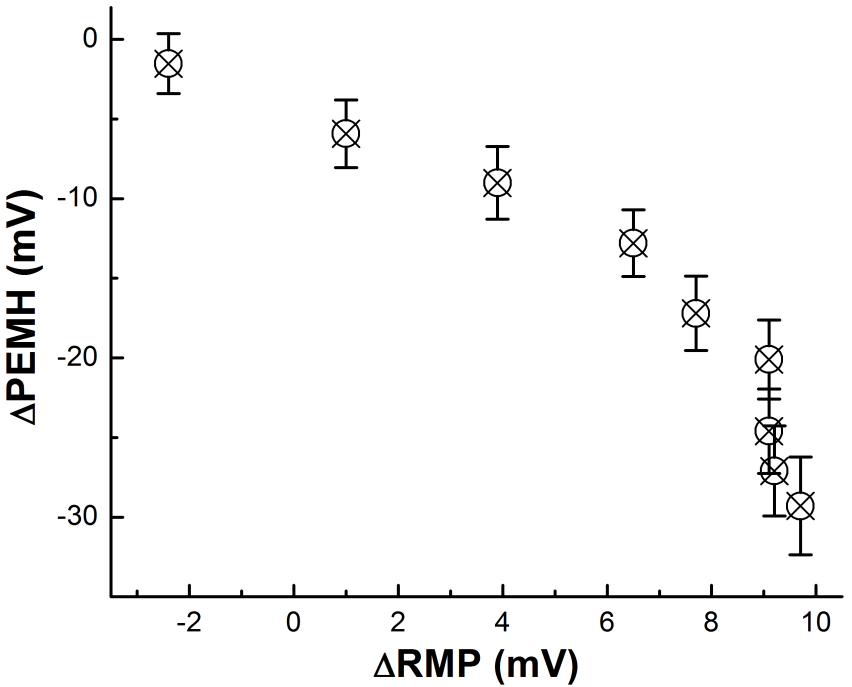


**4A**:

**X-axis**: clamp potential; **Y-axis**: difference between the peak PEMH and the clamp potential (ΔPEMH)

**4B**:

**X-axis**: difference between RMP and clamp potential (ΔRMP); **Y-axis**: ΔPEMH

Average data were presented as mean ± 1SD, *n* = 5 complete sets of recordings.
