# Supplementary material for: Remodeling of Hyperpolarization-Activated Current, Ih, in Ah-Type Visceral Ganglion Neurons Following Ovariectomy in Adult Rats
Source: PLoS One. 2013 Aug 12;8(8):e71184. doi: 10.1371/journal.pone.0071184 (PMC3741359; doi:10.1371/journal.pone.0071184)
Supplement: Table S1 — Electrical properties of myelinated Ah-type neurons in intact slices from control (no surgery), ovariectomized (OVX) and sham-operated adult rats. (DOCX) [file pone.0071184.s005.docx]

**Table S1:** Electrical properties of myelinated Ah-type neurons in intact slices from control (no surgery), ovariectomized (OVX) and sham-operated adult rats.

| **Parameter** |  | **Myelinated Ah-type nodose neurons** | | |
| --- | --- | --- | --- | --- |
|  | ANOVA | Control, *n* = 10 | OVX, *n* = 9 | Sham, *n* = 7 |
| **CV, m/s** |  | 11.8 ± 3.4 | 9.8 ± 2.2 | 13.3 ± 4.5 |
| **RMP, mV** |  | 63.0 ± 1.0 | 63.8 ± 1.2 | 62.7 ± 1.5 |
| **APFT, mV** |  | 37.0 ± 2.9 | 35.3 ± 3.2 | 38.4 ± 3.5 |
| **APFF Hz** | < 0.01 | 23.3 ± 3.5 | 2.6 ± 0.51**^††^ | 20.3 ± 3.9 |
| **AP_Peak,_ mV** |  | 55.5 ± 3.4 | 53.1 ± 3.5 | 56.6 ± 4.1 |
| **APD, ms** | < 0.01 | 1.92 ± 0.3 | 1.36 ± 0.5**^††^ | 1.89 ± 0.52 |
| **AHP_Peak_, mV** |  | -66.6 ± 1.2 | -65.4 ± 1.4 | -65.4 ± 1.9 |
| **AHP_80_, ms** |  | 29.4 ± 9.6 | 23.0 ± 12 | 22.4 ± 14 |
| **UV_APD50_, V/s** |  | 129 ± 9 | 136 ± 13 | 131 ± 15 |
| **DV_APD50_, V/s** | < 0.05 | 52.6 ± 6.2 | 65.6 ± 7.3*^†^ | 53.2 ± 5.5 |

Single APs were elicited by 2-ms depolarizing currents. Action potential firing frequency was assessed by applying step depolarizing currents of 1000-ms duration. Data are mean ± 1 SD. **P* < 0.05 and ***P* < 0.01 vs control, ^†^*P* < 0.05 and ^††^*P* < 0.01 vs sham. CV, conduction velocity; RMP, resting membrane potential; APFT, action potential firing threshold; APFF, action potential firing frequency; AP_Peak_, action potential peak; APD_50_, AP duration at 50% deflection; AHP_Peak_, peak afterhyperpolarization; AHP_80_, time for recovery to within 80% of RMP from AHP_Peak_; UV_APD50_, upstroke velocity as measured at APD_50_; DV_APD50_, downstroke velocity as measured at APD_50_; *n* indicates the number of cells. The *t*-test with Bonferroni correction was used for *post hoc* analyses.
